# Effective management of large renal stones using retrograde intrarenal surgery with combined direct in-scope suction and flexible and navigable ureteral access sheath: a case report

**DOI:** 10.3389/fruro.2025.1634754

**Published:** 2025-10-24

**Authors:** Nadhif Faza Ananda, Karen Graciana Setiawan, Vindasya Almeira, Faiza Lavina Meutia, Favian Ariiq Rahmat, Armand Achmadsyah, Nur Rasyid, Widi Atmoko, Ponco Birowo

**Affiliations:** ^1^ Faculty of Medicine, University of Indonesia, Depok, Indonesia; ^2^ Department of Urology, Faculty of Medicine, University of Indonesia - dr. Cipto Mangunkusumo General Hospital, Jakarta, Indonesia

**Keywords:** renal stone, thulium laser, stone-free rate, DISS fibre, RIRS- retrograde intrarenal surgery

## Abstract

**Introduction:**

Managing large renal stones with minimally invasive techniques is challenging, particularly in achieving optimal stone clearance. This report highlights the use of Retrograde Intrarenal Surgery (RIRS) combined with Combined Direct In-Scope Suction (DISS), Flexible and Navigable Ureteral Access Sheath (FANS), and thulium laser as an innovative approach to managing a complex staghorn stone.

**Materials and method:**

A 39-year-old male presented with left flank pain and hematuria for three weeks. Imaging revealed a large staghorn stone in the left kidney (2.8 x 1.5 x 4.3 cm, 1000–1200 HU) alongside smaller stones (0.5–1 cm) with associated grade II hydronephrosis and suspected ureterovesical junction stricture. RIRS was performed with a thulium laser and DISS and FANS to optimize visualization and enable efficient stone debris removal. Postoperative imaging revealed a single residual fragment (10 x 7 mm), resulting in a stone-free rate of 96.2%.

**Discussion:**

The integration of DISS and FANS in RIRS enhances procedural efficacy by maintaining a clear field of view and facilitating real-time removal of stone fragments. This approach proved to be effective in managing a large renal stone with minimal invasiveness, offering advantages such as reduced operative challenges and improved outcomes. This technique demonstrates the potential for RIRS as a viable alternative in selected scenarios.

**Conclusion:**

RIRS combined with DISS and FANS represents a promising method for managing complex renal stones, achieving high stone-free rates with minimal complications.

## Introduction

Managing large renal stones continues to be a significant challenge in urology, requiring effective yet minimally invasive approaches. While in common cases, percutaneous nephrolithotomy (PCNL) is the gold standard for stones larger than 2 cm due to its high stone-free rates, this often associated with increased risks of bleeding, infection, and adjacent organ damage (1, 2). These limitations have driven advancements in retrograde intrarenal surgery (RIRS), which supported by innovations such as flexible ureteroscopes and high-power lasers, has gained traction as a safer alternative in select cases (3, 4).

However, RIRS for large stones has inherent challenges, including poor visibility caused by the “snow globe” effect, incomplete fragment clearance, and elevated intrarenal pressure during lithotripsy, which may lead to complications. 5 To address these issues, suction-assisted technologies such as Direct In-Scope Suction (DISS) and Flexible Navigable Ureteral Access Sheaths (FANS) have been developed. These devices not only improve visibility and fragment clearance but also reduce intrarenal pressure, thereby minimizing complications (3, 4).

This report presents a case of successful management of a large staghorn stone using RIRS combined with DISS, FANS, and thulium laser lithotripsy. It highlights how these technologies enhance the efficacy and safety of RIRS, demonstrating their potential as an alternative for managing complex renal stones. Written informed consent was obtained from the patient for publication of this case report and any accompanying images. A copy of the written consent is available for review by the Editor-in-Chief ofthis journal. In addition, we declare that our case report also already compliant with SCARE Checklist which the detail is submitted during publicational process (5).

## Presentation of case

**Figure 1:**
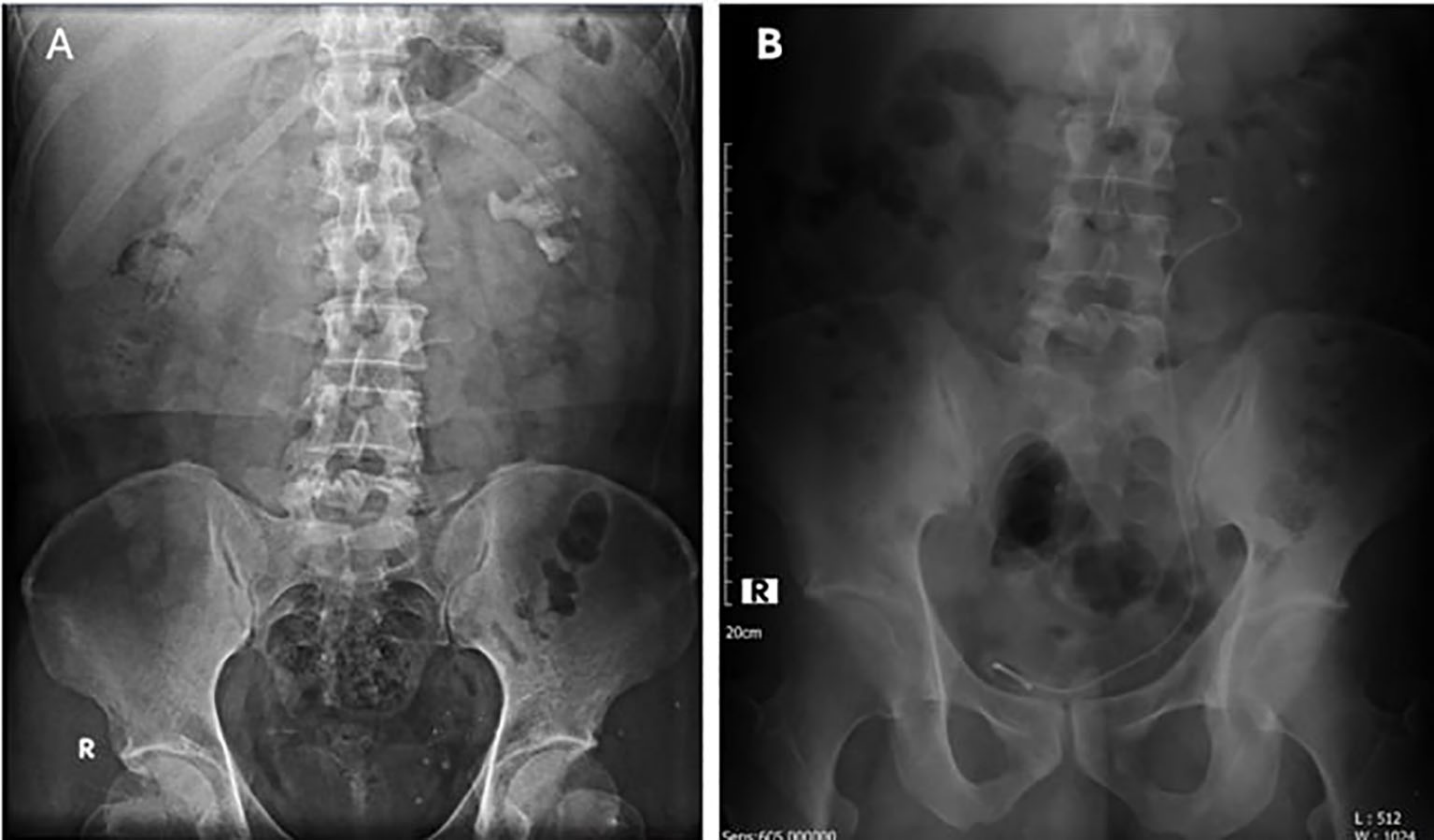
**(A)** Preoperative X-ray showing a staghorn stone in the left kidney; **(B)** Postoperative X-ray after RIRS with DISS, showing a DJ stent and a residual fragment (10 x 7 mm).

A 39-year-old male presented with colicky flank pain persisting for three weeks before entering hospital. His past medical history is reported to have dyslipidemia and gout. However, there is no past surgical procedure history. Patient confirm to experience a prior episode of spontaneous stone passage. Initial evaluation with urinalysis revealed microscopic hematuria (+1). Ultrasonography confirmed the presence of a large renal stone, prompting further diagnostic imaging. An X-ray (**Figure 1A**) and non-contrast computed tomography (CT) scan identified multiple left nephrolithiasis, including a staghorn stone measuring approximately 2.8 × 1.5 × 4.3 cm with a density of 1000–1200 Hounsfield Units (HU), occupying the mid-inferior pelvicalyceal system. Additional smaller stones (0.5–1 cm) were noted in the mid-inferior calyces. Associated findings included grade II hydronephrosis and left-sided hydroureter extending from the proximal to the distal ureter. A suspected ureterovesical junction (UVJ) stricture was also observed, likely secondary to the previous stone passage. No stones were identified in the ureter.

Initial management included the administration of potassium-magnesium citrate, ciprofloxacin, and ketorolac to alleviate symptoms and support renal function. Preoperative evaluation revealed normal laboratory parameters: hemoglobin 13.7 g/dL, hematocrit 41.6%, leukocyte count 4.6 × 10^9^/L, and platelet count 352 × 10^9^/L. Renal function tests showed blood urea 35 mg/dL, creatinine 1.21 mg/dL, and an estimated glomerular filtration rate (eGFR) of 78 mL/min/1.73m². Electrolyte levels were within normal ranges (Na/K/Cl 134/3.8/103 mmol/L), and liver enzymes were slightly elevated (SGOT/SGPT 26/48 U/L). Coagulation profile and lipid panel were unremarkable.

The patient was deemed fit for surgery by internal medicine and anesthesiology teams. He subsequently underwent RIRS with the insertion of a double-J (DJ) stent. The procedure utilized a ureteral access sheath (UAS) and a flexible ureteroscope (FURS) equipped with Direct In-Scope Suction (DISS), along with Flexible Antegrade Nephroscopy (FANS) for enhanced visualization and stone retrieval. A thulium laser was employed for stone fragmentation with settings of 1.5 J at 15 Hz, delivering a total power of 22.5 W. The total operative time was 170 minutes (skin-to-skin), with a laser activation time of 95 minutes, delivering an estimated total energy of approximately 125 kJ. Continuous irrigation under suction assistance totaled approximately 11.5 L. Estimated blood loss was 30 mL, and no transfusion was required.

## Result

**Figure 2:**
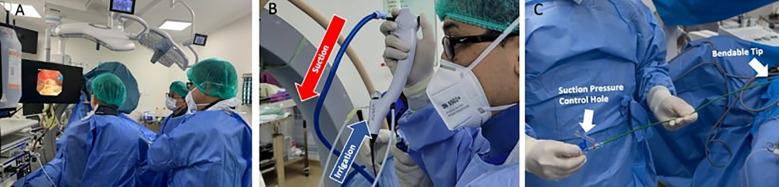
**(A)** Intraoperative view during RIRS; **(B)** Direct In-Scope Suction device used; **(C)** Flexible and Navigable Ureteral Access Sheath device used.

**Figure 3:**
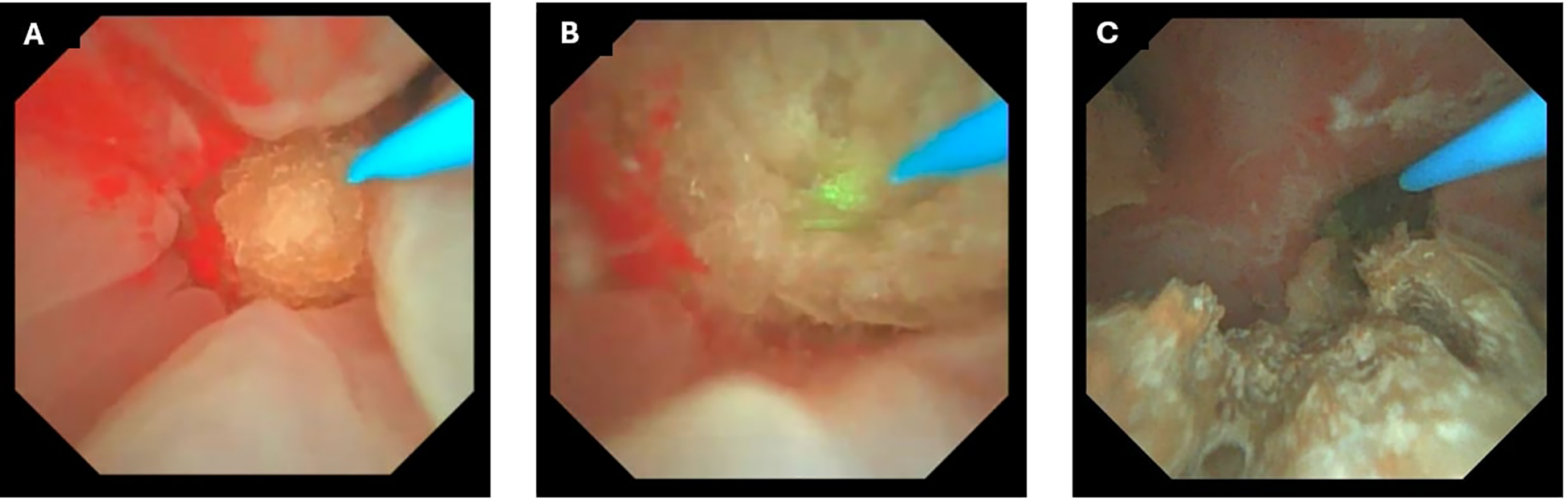
Intraoperative endoscopic views during retrograde intrarenal surgery (RIRS) with thulium fiber laser, Direct In-Scope Suction (DISS), and Flexible and Navigable Ureteral Access Sheath (FANS). **(A)** Initial visualization of a large staghorn stone before initiation of laser dusting and fragmentation. **(B)** Laser dusting in progress using thulium fiber laser, with visible ablation effect on the stone surface. **(C)** Intraoperative view following partial fragmentation, showing residual stone debris within the renal calyx.

Intraoperatively (**Figure 2**), the incorporation of DISS and FANS provided significant advantages. The DISS system ensured a consistently clear surgical field, minimizing the need for irrigation and improving the accuracy of laser application. The FANS allowed for precise navigation of the renal calyces, enabling efficient fragmentation and retrieval of stone fragments (**Figure 3**). These advancements made the procedure remarkably straightforward, even for a large and complex staghorn stone. The surgery was completed within three hours, showcasing the efficiency and reliability of this technique. Quantitative intraoperative metrics are summarized in **Table 1**, including total operative time (170 minutes), laser activation time (95 minutes), irrigation fluid volume (11.5 L), and estimated blood loss (30 mL). 

**Table 1:** Key operative metrics for the present case.

Metric	Value
Total operative time	170 minutes
Laser activation time (TFL)	95 minutes
Estimated total laser energy	~125 kJ
Irrigation fluid volume	11.5 L
Estimated blood loss	30 mL
Transfusion required	No

Postoperatively, the patient reported mild dysuria and reddish urine discoloration, which resolved within 24 hours. A Foley catheter was maintained for immediate postoperative management. Follow-up imaging with plain abdominal X-ray (**Figure 1B**) confirmed proper placement of the DJ stent, with the proximal tip at the L4-L5 vertebral level and the distal tip within the pelvic cavity. A single residual stone fragment measuring 10 × 7 mm was noted at the L3 vertebral level, resulting in a stone-free rate of 96.2%.

Laboratory results postoperatively showed hemoglobin 13.2 g/dL, indicating a minimal decline from preoperative levels (0.5 g/dL reduction). Renal function remained stable, with creatinine at 1.18 mg/dL and an eGFR of 79 mL/min/1.73m². Electrolytes (Na/K/Cl 134/3.7/104 mmol/L) were also within normal limits. These findings indicate that the procedure was well-tolerated, with no significant renal impairment, electrolyte disturbances, or excessive blood loss.

On postoperative day two, the patient was discharged in stable condition. He was advised to maintain hydration, continue potassium-magnesium citrate, and attend a follow-up appointment in two weeks for clinical assessment and possible removal of the DJ stent.

He was also counseled on dietary modifications to prevent recurrence and educated on recognizing signs of potential complications, such as fever, persistent pain, or difficulty urinating. At discharge, the patient expressed satisfaction with the care provided and had no complaints. Patients explained their satisfaction in terms no significant scar being formed and symptoms could be resolved in just one surgical operation. He also reported satisfaction with the minimally invasive approach and expressed relief at avoiding percutaneous surgery.

## Discussion

### Implementation

The management of complex renal stones, particularly staghorn calculi larger than 2 cm, presents significant challenges due to risks such as hydronephrosis, infection, and recurrence. Gold standard deemed achieving stone-free rates (SFRs) as high as 78–95% for large stones (6). However, this carries the risks of complications (7%), including bleeding and adjacent organ injury, along with longer recovery times, driving the adoption of minimally invasive alternatives like retrograde intrarenal surgery (RIRS) (7, 8).

In this case, a 2.8 × 1.5 × 4.3 cm staghorn stone with moderate hydronephrosis and a suspected ureterovesical junction (UVJ) stricture was successfully treated using RIRS in conjunction with direct in-scope suction (DISS), a flexible and navigable ureteral access sheath (FANS), and a thulium fiber laser (TFL). Postoperative imaging confirmed an SFR of 96.2%, aligning with previous study that report SFRs between 85–96% for RIRS using advanced devices. 2 The DISS system was pivotal in maintaining intraoperative clarity by actively removing stone fragments and debris, significantly reducing operative time. In a recent study, DISS have a quick mean operation time of 50 minutes (9).

The use of Thulium fiber laser (TFL) added a significant advantage in this case. TFL has been reported to achieve higher ablation rates than conventional Holmium: YAG (Ho: YAG) lasers, with earlier studies suggesting up to a 1.5–4-fold increase in stone fragmentation efficiency. 10 Nevertheless, recent systematic reviews indicate that when Ho: YAG is combined with pulse modulation technologies such as Moses, ablation efficiency between the two platforms becomes largely comparable, thereby underscoring that the principal clinical advantage of TFL is not enhanced fragmentation alone (11). Instead, TFL demonstrates a consistently lower retropulsion profile, with prospective studies showing Likert scale retropulsion scores of 0–1 in approximately 85% of cases, compared with substantially higher scores using Ho: YAG (10).

In addition, emerging evidence supports the use of suctioning ureteral access sheaths (S- UAS), including novel platforms such as FANS with DISS, which have been shown to improve stone-free rates, reduce postoperative infectious complications, and shorten hospitalization without prolonging operative time. Taken together, these data suggest that combining TFL with suctioning technologies may represent a synergistic strategy for the management of large stone burdens which providing efficient fragmentation with superior intraoperative control and safety (12). This comparison is summarized in **Table 2**, which contrasts the clinical outcomes and operative characteristics of PCNL and RIRS augmented with DISS/FANS.

**Table 2:** Comparison of PCNL vs RIRS with DISS/FANS for large or complex renal stones.

Feature/outcome	PCNL (standard/mini)	RIRS + DISS/FANS (present case & literature)
Typical indication	Stones ≥2 cm, staghorn calculi (Rassweiler et al., 2000; BJU Int. 86:919-28)	Selected ≥2 cm/staghorn when advanced devices and expertise are available (Geavlete et al., J Clin Med. 2024;13:2493)
Stone-free rate (early)	80–95% depending on technique and stone size (Rassweiler et al., 2000)	85–96% in suction- assisted RIRS series; 96.2% in present case (Nedbal et al., World J Urol. 2024;42:560)
Total operative time	~70–100 min for typical large stone	~60–90 min in series; 170 min in present case due to staghorn + UVJ stricture
Laser activation time	Not applicable (pneumatic/ultrasonic lithotriptor)	95 minutes (present case; TFL)
Blood loss/transfusion	Higher EBL; transfusion 1–6% (Giulioni et al., Actas Urol Esp. 2024;48:57-70)	Minimal (~30 mL), transfusion rare
Hospital stay	2–4 days (often longer for complex cases)	≤48 hours in many RIRS cases; 2 days in present case
Complications	Bleeding, adjacent organ injury, infection	Lower bleeding risk; infectious risk mitigated by suction & IRP control (Gauhar et al., Eur Urol Focus. 2024)
Intrarenal pressure (IRP)	Not relevant	Suction maintains IRP ~20–40 mmHg, reducing pyelovenous backflow
Equipment	Tract dilation, nephroscope, lithotriptor	Flexible ureteroscope + TFL + DISS + FANS

The Flexible and Navigable Ureteral Access Sheath (FANS) was another key component in this procedure. FANS offers significant advantages over traditional ureteral access sheaths by allowing improved maneuverability and access to challenging intrarenal locations. Its design enables navigation through tortuous calyces, facilitating complete visualization and efficient fragment clearance. Yue et al. demonstrated that navigable sheaths like FANS improved stone clearance rates to 76.3%, reduced complication rates to 9.9% (compared to 22.4% for traditional access sheaths), and shortened operative times to 56.5 minutes. Yue et al. demonstrated that navigable sheaths like FANS improved stone clearance rates to 76.3%, reduced complication rates to 9.9% (compared to 22.4% for traditional access sheaths), and shortened operative times to 56.5 minutes (13). In this case, FANS was instrumental in addressing residual fragments in hard-to-reach calyces, optimizing the outcome further.

### Advantages

This combined method offering advantages such as reduced operative challenges and improved outcomes. In this case, despite the complexity of a staghorn stone with a suspected UVJ stricture, the procedure was completed in 170 minutes with a laser activation time of 95 minutes, maintaining clear visualization throughout with suction- assisted irrigation totaling 11.5 L and minimal blood loss (30 mL). These metrics align with reported ranges for suction-assisted RIRS in complex stone cases, underscoring the safety and feasibility of this approach.

### Limitation

Despite all the advantages, residual fragments (10 × 7 mm initially) were noted postoperatively, although they were subsequently reduced to 2–4 mm during follow-up. Residual fragments are common with a median volume of 63.8 of residual cases involving stones larger than 2 cm, necessitating vigilant follow-up to prevent recurrence (14).

Additionally, the specialized equipment and skills required for DISS, TFL, and FANS limit their widespread availability (15, 16).

This adoption procedures also poses several challenges in terms of cost, training, and accessibility. The device itself requires an investment of approximately IDR 3.9 million for FANS and IDR 15 million for DISS, with a total procedural cost including hospital net acquisition (HNA, the official net acquisition cost of medical devices in Indonesia) and value-added tax (VAT/PPN) reaching around IDR 15 million. While these expenses may be balanced by reduced retreatment rates and shorter hospital stays, the upfront cost remains a barrier, especially in lower-resource settings. Moreover, adequate training through structured workshops and supervised clinical practice is essential to ensure safety and efficacy. Limited availability and high procurement costs in rural hospitals is still considered constrained and may further restrict access, underscoring the need for shared equipment models, subsidized programs, or integration into national training CURRICULA to enhance equity of care.

### Troubleshooting and challenges

In this case, the most relevant intraoperative challenge was surgeon fatigue due to the prolonged operative time required for managing a large staghorn stone. The procedure involved extensive endoscopic manipulation, which resulted in wrist stiffness, thumb discomfort, and shoulder strain. These symptoms, although not compromising the surgical outcome, highlight the ergonomic burden of lengthy RIRS procedures and underline the importance of optimizing operative efficiency.

From a device-related perspective, a specific limitation of the DISS system is its finite operating lifespan, as the unit is designed to automatically shut down after approximately four hours of continuous use. While this did not directly affect the present case, it remains a practical concern for very complex procedures and should be taken into account when planning operative strategies. In addition, the high volume of irrigation fluid associated with suction use requires careful monitoring of intrarenal pressure and perioperative fluid balance. These limitations emphasize the need for ongoing refinement of both device design and perioperative protocols.

### Future direction

Nevertheless, future directions for RIRS with advanced tools like DISS, FANS, and TFL is still promising and could complementing gold standard in terms of outcome, especially for post- procedure evaluation. Then, further research to assess its efficacy is considered important, such as multicenter randomized trials comparing outcomes from gold standard for complex renal stones. Standardizing protocols for laser settings, suction systems, and navigable sheath designs could further enhance procedural success rates. This case highlights the transformative potential of integrating RIRS which offering highly effective approach for managing complex renal stones (17, 18).

## Conclusion

In conclusion, the integration of DISS and FANS in RIRS represents a paradigm shift in the management of complex renal stones. By combining efficient suction, precise navigation, and effective fragment management, this approach provides a minimally invasive yet highly effective alternative approach. Further studies should explore long-term outcomes and cost-effectiveness to establish its place as a standard of care in modern endourology.

## Data Availability

The raw data supporting the conclusions of this article will be made available by the authors, without undue reservation.
